# Socioeconomic factors affecting psychotherapy use rates

**DOI:** 10.1093/eurpub/ckac129.684

**Published:** 2022-10-25

**Authors:** S Selinheimo, K Gluschkoff, J Turunen, J Kausto, A Väänänen

**Affiliations:** Finnish Institute of Occupational Health, Helsinki, Finland

## Abstract

**Objectives:**

Previous studies indicate socioeconomic inequalities in psychotherapy utilization. The aim of this study was to assess the associations of individual annual incomes with the utilization of long-term rehabilitative psychotherapy during nine-year follow-up in men and women. As secondary analyses we assessed the association of main activity with the utilization of psychotherapy.

**Methods:**

For this study, we selected those from a random sample of the working-age population (18-55 years) with information about income at each time point during the follow-up from 2011 to 2019 (N = 736 613). Psychotherapy usa during the follow-up period served as dependent variable and sosiodemographic variables, annual incomes and main activity (employed, unemployed, studying, other) were used as independent variables. To examine change in the psychotherapy use rates over time, we used sex-stratified generalized estimating equations logistic regression models with predicted marginal probabilities.

**Results:**

Psychotherapy use rate was constantly higher among women than in men (in 2011 0.8% and 0.2%) and increased from 2011 to 2019 among both genders and income quartiles (among women 174% - 231% and among men 213% - 248% increase between quartiles). Among men, psychotherapy use rate was highest among lowest income quartile throughout the study interval. Among women such difference was not observed. Among women, studentś psychotherapy use increased significantly when compared to other groups from 2011 to 2019 (299% increase vs 89% - 210% increase among other groups). A similar pattern was seen among studying men versus other groups.

**Conclusions:**

Between 2011 and 2019 the probability of having psychotherapy increased among both genders. Unexpectedly, pro-rich psychotherapy use rate was not observed. The highest probability to use psychotherapy in lowest income quartile might be linked with differences in health care systems for students and for other.

